# Deregulated Renal Calcium and Phosphate Transport during Experimental Kidney Failure

**DOI:** 10.1371/journal.pone.0142510

**Published:** 2015-11-13

**Authors:** Wilco P. Pulskens, Melissa Verkaik, Fareeba Sheedfar, Ellen P. van Loon, Bart van de Sluis, Mark G. Vervloet, Joost G. Hoenderop, René J. Bindels, NIGRAM Consortium

**Affiliations:** 1 Dept. of Physiology, Radboud University Medical Center, Nijmegen, The Netherlands; 2 Dept. of Nephrology, Radboud University Medical Center, Nijmegen, The Netherlands; 3 Dept. of Nephrology, VU University Medical Center, Amsterdam, The Netherlands; 4 Dept. of Pediatrics, Molecular Genetics Section, University Medical Center Groningen, Groningen, The Netherlands; University Medical Center Utrecht, NETHERLANDS

## Abstract

Impaired mineral homeostasis and inflammation are hallmarks of chronic kidney disease (CKD), yet the underlying mechanisms of electrolyte regulation during CKD are still unclear. Here, we applied two different murine models, partial nephrectomy and adenine-enriched dietary intervention, to induce kidney failure and to investigate the subsequent impact on systemic and local renal factors involved in Ca^2+^ and P_i_ regulation. Our results demonstrated that both experimental models induce features of CKD, as reflected by uremia, and elevated renal neutrophil gelatinase-associated lipocalin (NGAL) expression. In our model kidney failure was associated with polyuria, hypercalcemia and elevated urinary Ca^2+^ excretion. In accordance, CKD augmented systemic PTH and affected the FGF23-αklotho-vitamin-D axis by elevating circulatory FGF23 levels and reducing renal αklotho expression. Interestingly, renal FGF23 expression was also induced by inflammatory stimuli directly. Renal expression of Cyp27b1, but not Cyp24a1, and blood levels of 1,25-dihydroxy vitamin D_3_ were significantly elevated in both models. Furthermore, kidney failure was characterized by enhanced renal expression of the transient receptor potential cation channel subfamily V member 5 (TRPV5), calbindin-D_28k_, and sodium-dependent P_i_ transporter type 2b (NaP_i_2b), whereas the renal expression of sodium-dependent P_i_ transporter type 2a (NaP_i_2a) and type 3 (PIT2) were reduced. Together, our data indicates two different models of experimental kidney failure comparably associate with disturbed FGF23-αklotho-vitamin-D signalling and a deregulated electrolyte homeostasis. Moreover, this study identifies local tubular, possibly inflammation- or PTH- and/or FGF23-associated, adaptive mechanisms, impacting on Ca^2+^/P_i_ homeostasis, hence enabling new opportunities to target electrolyte disturbances that emerge as a consequence of CKD development.

## Introduction

Chronic kidney disease (CKD) is a major public health issue with a high prevalence currently affecting millions of people worldwide [1]. CKD results in a significant morbidity and mortality and highly associates with the development of cardiovascular events, the primary cause of death in CKD patients [2–4]. A prominent hallmark of CKD that may contribute to this increased risk is a disturbed electrolyte homeostasis, including calcium (Ca^2+^) and phosphorus (P_i_) deregulation [5]. Physiological Ca^2+^ and P_i_ levels in the blood are maintained by complex endocrine systems comprising the parathyroid hormone (PTH) and phosphaturic hormone Fibroblast Growth Factor 23 (FGF23) and vitamin D system [6–8]. PTH and FGF23 inhibit renal P_i_ reabsorption and are the primary regulators of renal 1,25-dihydroxy vitamin D_3_ (active vitamin-D) synthesis. 1,25-dihydroxy vitamin D_3_ itself also regulates urinary calcium excretion by the kidney [6–8]_._

In addition to bone-derived FGF23, other cell types may contribute to circulating FGF23 levels, including fibroblasts and smooth muscle cells [9]. Moreover, several neuroendocrinologic factors and cytokines directly influence FGF23 secretion into the circulatory system [10, 11]. To enable downstream signalling, FGF23 requires membrane-bound αklotho as its obligate co-receptor [12, 13]. αklotho is predominantly expressed in renal epithelium and the parathyroid gland, hence providing tissue-specificity for FGF23 signalling [14]. In addition, soluble αklotho present in the circulation or urine can regulate electrolyte transport, through the epithelial Ca^2+^ channel (TRPV5) [15] and the renal outer medullary potassium channel (ROMK1) [16–19]. There are well-described reductions in renal and parathyroid klotho in CKD. It has been shown [20] that CKD associates with reduced αklotho expression and that it is related to elevated FGF23 levels, in response to persistent phosphate retention [21–23]. In turn, this may lower systemic vitamin-D levels, which contributes to the pathogenesis of secondary hyperparathyroidism. The consequences of CKD-induced alterations in FGF23-αklotho-vitamin-D signalling on renal tubular electrolyte regulatory mechanisms are, however, still unclear. Hence, in the current study, we characterized the impact of two different experimental models of progressive kidney failure on FGF23-αklotho-vitamin-D signalling and local renal Ca^2+^ and P_i_ transport regulation. In addition we examined the consequences of inflammatory stimuli and the effect of FGF23 on several mineral-regulating channels on the tubular epithelium.

## Material and Methods

### Ethical statement

This study was carried out in strict compliance with the legal Dutch animal welfare act. All experimental procedures performed were approved by the animal ethics board of the Radboud University Nijmegen (permit-no: RU-DEC 2013–068 and RU-DEC 2013–224) or by VU University Medical Center (VUmc), Amsterdam (permit-no: Fys 12–01) or by University Medical Center Groningen (UMCG), Groningen (permit-no: 5321K and 6697C) and all efforts were made to minimize suffering of the animals. A completed ARRIVE guidelines checklist is included in S1 File.

### Mice

Pathogen-free eight to ten weeks old male C57Bl/6 mice were purchased from Charles River Laboratories and housed under standardized conditions in the animal facilities of the VU University Medical Center (VUmc), Amsterdam, Radboud University Medical Center, Nijmegen, and University Medical Center Groningen (UMCG), Groningen, The Netherlands. Transgenic mice expressing enhanced green fluorescent protein (EGFP) driven by the TRPV5 promoter (TRPV5-EGFP) were generated as described previously [24] and housed in the animal facility of the Radboud University Medical Center [25]. The presence of EGFP expression was genetically confirmed by routine PCR screening. All mice received water and food *ad libitum* and only age-matched male mice were used in this study. All experiments were approved and conducted following the guidelines of the local Animal Ethical Committees, adhering the guidelines of the European animal welfare.

### Partial (5/6) nephrectomy and urine collection

Prior to surgery a small volume of peripheral blood was collected into EDTA-coagulated microtainers (BD Biosciences, Breda, The Netherlands). Partial nephrectomy (5/6Nx; n = 9) was performed under standardized sterile conditions and induced as described before [26, 27]. Briefly, a small abdominal midline incision of the skin and muscles was made under general anaesthesia (isoflurane) and preoperative analgesia (Buprenorphin; Temgesic (Schering-Plough, Houten, The Netherlands), 0.05 mg/kg intramuscular). The left kidney was decapsulated after which both, the upper and lower pole were ablated by cauterization (High-temperature fine tip Cautery, Bovie Medical Corporation, Clearwater, FL, USA). Subsequently, the contralateral kidney was decapsulated and renal blood vessels and ureter were ligated, after which the entire kidney was removed. The abdomen was closed with sutures in two layers and all mice received subcutaneous injections of postoperative analgesia two days after surgery (Ketoprofen; Ketofen (Merial S.A.S., Velserbroek, The Netherlands), 5 mg/kg). Sham-operated mice were used as controls and underwent the similar protocol including decapsulation of both kidneys, except for the renal ablation (n = 5). After three weeks, mice were placed into individual metabolic cages (Tecniplast, Buguggiate, Italy) enabling 24-hours urine collection. Mice were sacrificed and blood was divided into EDTA- and heparin-coagulated microtainers and centrifuged for 10 minutes (min) at 1,500 g at 4°C. Plasma samples were stored at -80°C. Remnant renal tissue was isolated, subdivided into two equal parts and either directly snap-frozen into liquid nitrogen or fixated in 1% w/v paraformaldehyde-lysine-periodate (PLP) solution for 2 hours (hrs) at room temperature, subsequently incubated overnight at 4°C in phosphate buffered saline (PBS) containing 15% w/v sucrose before snap-frozen in liquid nitrogen.

### Adenine-enriched dietary intervention

Prior to the start of diet, a small volume of peripheral blood was isolated by cheek puncture into microvette tubes (Sarstedt, Etten-Leur, The Netherlands). Mice of about 25 gram body weight were fed with a 0.2% w/w adenine (Sigma-Aldrich, Saint-Louis, MO, USA; cat.no. A8626) containing diet (Bio Services; Uden, The Netherlands) during a period of either 2 or 4 weeks (n = 5/group). A control group (n = 5) was fed a standard diet and sacrificed after 4 weeks. Body weights were recorded every other day. After 2 or 4 weeks, mice were placed into individual metabolic cages (Tecniplast) for 24 hrs before sacrificed under general anaesthesia (isoflurane). Blood was isolated from the orbital sinus and collected in microvette tubes. Samples were allowed to clot for 2 hrs at room temperature and subsequently centrifuged 10 min at 9,000 g at 4°C to enable serum isolation, and subsequently stored at -80°C. Kidney tissue was isolated and processed as described above.

### Systemic administration of FGF23

Male TRPV5-EGFP mice of 5–7 weeks old received a single intravenous tail vein administration (IV) of 160 μg/kg Carrier-free recombinant mouse FGF23 (rFGF23; R&D Systems, Minneapolis, MN, USA; cat.no 2629-FG-CF) or vehicle (saline) in a total volume of 100 μl (n = 5/group). These mice were non-CKD mice and after 24 hrs were sacrificed under total anaesthesia (isoflurane). Blood and kidney were collected and processed, as described above.

### Systemic administration of Concanavalin A (ConA) and Tumor Necrosis Factor (TNF)

Male mice received a single intravenous retro-orbital administration of 20 ug/kg of bodyweight ConA or vehicle (PBS). A subset of mice received a single intravenous retro-orbital administration of 15 ug/kg of bodyweight TNF or vehicle. These mice were non-CKD mice and were sacrificed either after 8 hrs ConA administration or after 1 hr TNF administration. Kidney were collected and processed as described above.

### Electrolyte measurement

Systemic (using either EDTA-coagulated plasma from 5/6Nx mice or serum samples from ADE-treated mice) and urinary concentrations of urea, and P_i_ were determined by in-hospital services using automatic biochemical analyzers. Blood Ca^2+^ concentrations were determined in heparin-coagulated plasma (from 5/6Nx mice) or serum (from ADE treated mice) and in urinary samples, verified using a commercial serum standard (Precinorm U, Roche, Switzerland) and measured as described previously [28].

### Immunoassays

EDTA-coagulated plasma (from 5/6Nx mice) or serum (from ADE treated mice) samples were used in enzyme-linked immunosorbent assays (ELISA) to determine circulating levels of FGF 23 and PTH 1–84 (both Immutopics International). Heparin-coagulated plasma (from 5/6Nx mice) and serum (from ADE treated mice) samples were used to determine circulating 1,25-dihydroxy vitamin D_3_ (active vitamin-D) levels using a radioimmunoassay (Immunodiagnostic Systems; IDS, Liege, Belgium). All assays were performed according to the manufacturer’s protocols.

### Western blotting

Renal tissues were homogenized in homogenization buffer A (HbA; 20 mM Tris-HCl (pH 7.4), 5 mM MgCl_2_, 5 mM NaH_2_PO_4_, 1 mM EDTA, 80 mM sucrose, 1 mM Phenylmethylsulfonyl fluoride (PMSF), 1 μg/mL leupeptin, and 10 μg/mL pepstatin A). Total protein concentrations were determined using the Bio-Rad Protein Assay (Bio-Rad, Munich, Germany) and protein was solubilised by 30 min incubation at 37°C in Laemmli buffer with 0.1 M *Dithiothreitol* (DTT). Protein (25 μg) was separated on an 8% w/v SDS-PAGE gel and transferred to a PVDF-nitrocellulose membrane (Immobilon-P, Millipore Corporation, Bedford, MA). Blots were blocked with 5% w/v non-fat dry milk (NFDM) into Tris-buffered saline (TBS)-0.1% v/v Tween (TBS-T) and subsequently incubated overnight at 4°C with either anti αklotho KL1-fragment (1:2,500; no. KM2076, kindly provided by prof. dr. M. Kuro-O, Texas, USA), anti-GFP (1:5000; Sigma-Aldrich, Zwijndrecht, The Netherlands) or β-actin (1:10,000; Sigma, clone AC-15) antibodies in 1% w/v NFDM/TBS-T. After incubation with appropriate peroxyoxalate-conjugated secondary antibodies for 1 hr at room temperature, proteins were visualized by chemiluminescence (Pierce, Rockford, IL). Immunoreactive proteins were scanned using Chemidoc XRS (Bio-Rad, Veenendaal, The Netherlands) and intensities were analyzed with Adobe Photoshop 7.0 software.

### RNA isolation and quantitative real-time RT-PCR

Total kidney mRNA was extracted from snap-frozen kidney tissue using Trizol Reagent (Life Technologies; Bleiswijk, The Netherlands), according to the manufacturer’s guidelines. All RNA samples were quantified by using an ND-1,000 spectrophotometer (NanoDrop Technologies, Rockland, DE). Total RNA (1.5 μg) was converted into cDNA using random hexamers (Promega, Madison, WI, USA) and M-MLV reverse transcriptase (Life Technologies). Mouse gene mRNA expression was analyzed by real-time quantitative reverse-transcription (RT)-PCR performed on a Bio-Rad CFX96™ Real-Time qPCR or 7900HT system (Applied Biosystems, Warrington, UK), using Bio-Rad iQ™ SYBR® Green Supermix or Power SYBR Green Master Mix (Roche, Mannheim, Germany). In addition specific gene expression was normalized to housekeeping gene (hypoxanthine-guanine phosphoribosyl transferase; HPRT) and analyzed according the 2^^-ddCt^ method [29]. The mouse gene-specific primer sets used are listed in S1 Table.

### Immunohistochemistry

Immunohistochemical staining was performed on 5 μm cryosections of PLP-fixed renal tissue samples [30]. Sections were stained with guinea pig anti-TRPV5 antibodies (1:2,000; [30]) or mouse anti-calbindinD_28k_ antibodies (1:2,000; Sigma Aldrich, Saint-Louis, MO, USA), with concomitant tyramide signal amplification (TSA) enhancement (Perkin Elmer, Waltham, MA, USA) following standard procedures. For visualization, sections were subsequently incubated with appropriate Alexa-488-conjugated secondary antibodies (Sigma Aldrich). Fluorescent images were made using a Zeiss fluorescence microscope (Sliedrecht, The Netherlands) equipped with an AxioCam digital photo camera.

### Statistics

Data are presented as mean ± SEM. Differences between groups were analyzed using Mann-Whitney *U* tests and calculated using Graphpad Prism version 5.0 software. A *p*-value <0.05 was considered as statistically significant.

## Results

### Partial nephrectomy and adenine-enriched dietary treatment induced kidney failure

**Fig 1:**
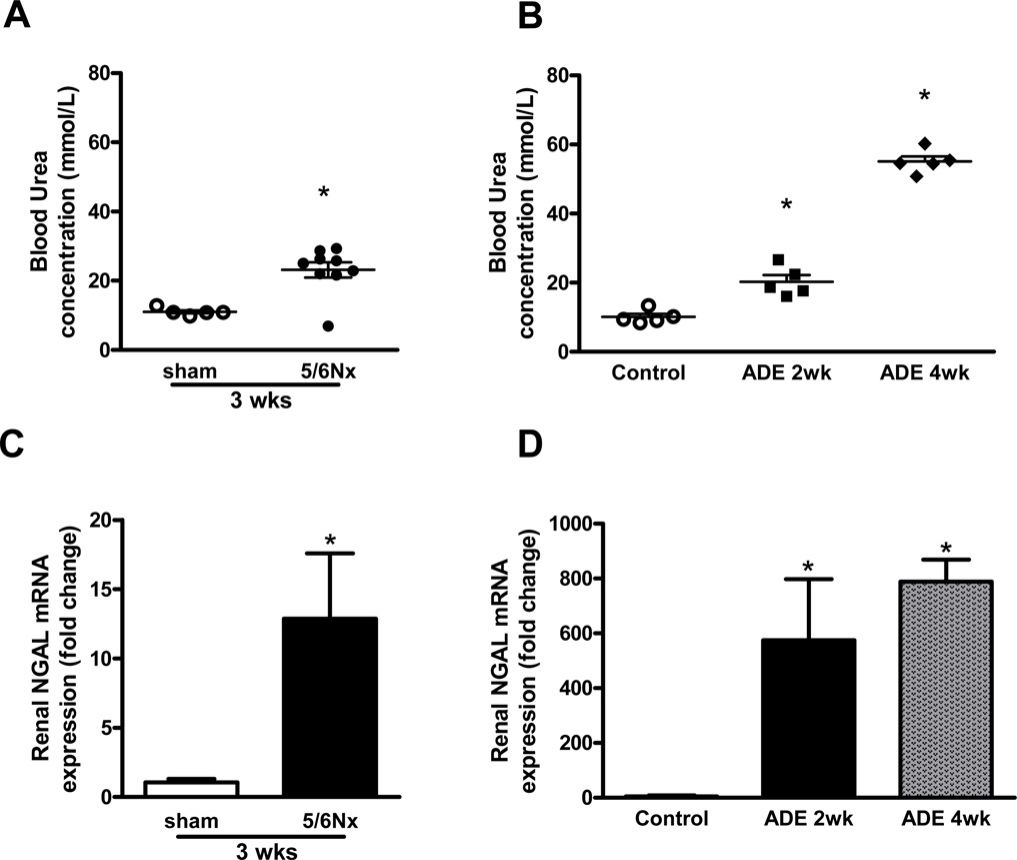
Partial nephrectomy and adenine-enriched dietary treatment induced CKD. Mice subjected to **(A)** partial nephrectomy (5/6Nx; n = 9) after 3 weeks and **(B)** adenine-enriched dietary treatment (ADE; n = 5) for either 2 or 4 weeks display elevated blood urea levels, when compared to sham-operated (n = 5) or control mice (n = 5), respectively. Renal expression of tubular injury marker NGAL is significantly elevated in **(C)** 5/6Nx mice and **(D)** ADE mice after 2 and 4 weeks, compared to sham-operated or control mice, respectively. Data are mean ± SEM. *: p<0.05 compared to either sham-operated or control mice.

The experimental models of partial nephrectomy (5/6Nx) and adenine-enriched dietary (ADE) treatment both induced renal failure. This is reflected by elevated blood urea levels in 5/6Nx mice compared to sham-operated mice after 3 weeks (Fig 1A) and the progressive uraemia observed in ADE mice when compared to control mice (Fig 1B). In addition, renal mRNA expression of tubular injury marker neutrophil gelatinase-associated lipocalin (NGAL) was increased 12-fold in kidney tissue of 5/6Nx mice and >500-fold in ADE-treated mice (Fig 1C and 1D). Moreover, mice subjected to either 5/6Nx or ADE treatment developed severe polyuria compared to sham-operated or control mice, respectively (Table 1).

**Table 1:** The effects of induced CKD on general physiological parameters.

	Water intake (mL)	Urine (mL)	Food intake (gr)	Faeces production (gr)
**Sham (n = 5)**	4.2±0.8	0.5±0.1	3.3±0.1	0.9±0.1
**5/6Nx (n = 9)**	8.4±1.0 *	2.5±0.2 *	3.2±0.3	0.9±0.1
**Ctrl diet (n = 5)**	4.4±0.3	1.3±0.2	3.9±0.1	2.0±0.1
**ADE 2wk (n = 5)**	10.8±0.5 *	6.0±0.2 *	3.8±0.1	1.7±0.1
**ADE 4wk (n = 5)**	11.1±0.4 *	7.9±0.4 *	3.4±0.1 *	1.4±0.1 *

Physiological measurements of mice subjected to 5/6Nx or sham operation, and subjected to ADE treatment for either 2 or 4 weeks or control diet. Data are collected after 24 hours housing in individual metabolic cages, and are indicated as mean ± SEM.

*: p<0.05 compared to either sham-operation or to control diet.

### Development of kidney failure disturbed electrolyte homeostasis

To investigate the consequences of induced renal failure on electrolyte homeostasis, blood levels and urinary excretion of Ca^2+^ and P_i_ were determined. No variations in blood Ca^2+^ and P_i_ levels were found within experimental groups prior to onset of the experiments (data not shown). 5/6Nx mice were hypercalcemic compared to sham-operated mice and additionally displayed elevated 24-hours urinary Ca^2+^ excretion (Table 2). In contrast, no differences were observed in blood P_i_ levels or 24-hours urinary P_i_ excretion. In accordance, ADE-treated mice displayed progressive hypercalcemia and increased urinary Ca^2+^ excretion when compared to control mice (Table 2). Blood P_i_ levels progressively increased and were significantly elevated after 4 weeks compared to control mice. No effects were detected on 24-hours urinary P_i_ excretion, whereas after 4 weeks ADE diet the fractional excretion of P_i_ was significantly elevated compared to control mice (26±2 and 40±3% Fractional Excretion of P_i_) for control and ADE-treated mice, respectively. In addition, blood Creatinine levels were significantly elevated in both models while urine Creatinine levels were significantly reduced only in 5/6Nx mice (Table 2).

**Table 2:** The effects of induced CKD on deregulated Ca^2+^ and P_i_ homeostasis.

	Creatinine (mmol/L)	Ca^2+^ (mmol/L)	P_i_ (mmol/L)	Urinary Crea (mmol/L)	Urinary Ca^2+^ (μmol/24hr)	Urinary P_i_
						(μmol/24hr)
**Sham (n = 5)**	21.6±1.2	1.7±0.1	1.7±0.0	1.9±0.2	2.0±0.3	28±2
**5/6Nx (n = 9)**	33.6±4.7 *	2.1±0.1 *	1.7±0.2	0.3±0.0 *	6.8±0.3 *	34±2
**Ctrl diet (n = 5)**	23.8±0.2	2.2±0.0	2.0±0.1	3.2±0.3	3.4±0.5	69±10
**ADE2wk (n = 5)**	27.2±1.2 *	2.4±0.1	2.1±0.1	3.2±0.4	9.3±0.7 *	83±13
**ADE 4wk (n = 5)**	53.6±0.9 *	2.8±0.0 *	2.7±0.2 *	3.8±0.1	18.7±1.2 *	58±8

Levels of creatinine, Ca^2+^ and P_i_ measured in blood or urine of sham-operated and 5/6Nx mice, or mice subjected to control diet or ADE treatment for either 2 or 4 weeks. Data are indicated as mean ± SEM.

*: p<0.05 compared to either sham operation or to control diet.

### Induction of experimental CKD altered FGF23-αklotho signalling

**Fig 2:**
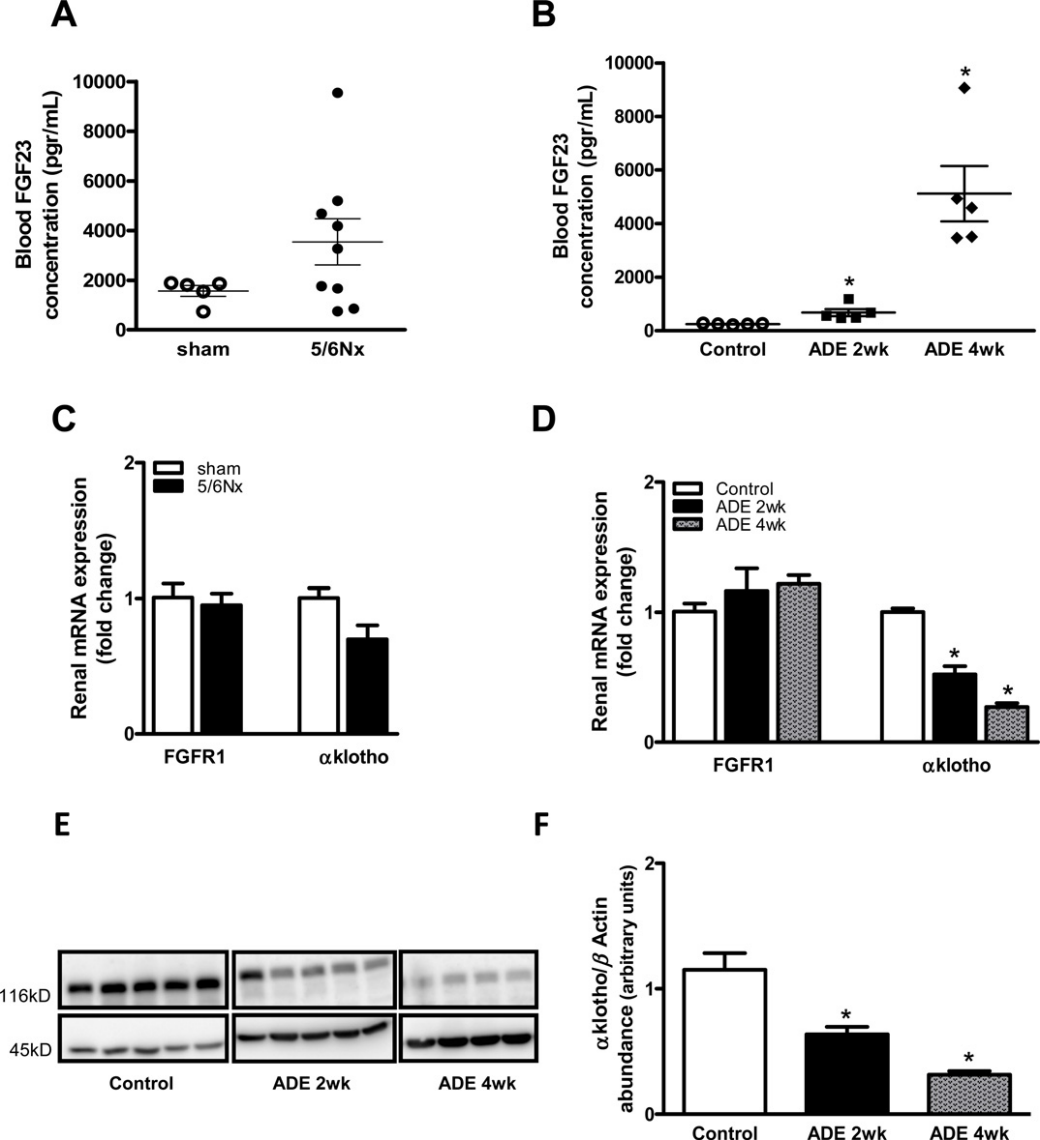
Progression of CKD altered FGF23-αklotho signalling. Experimental CKD deregulates FGF23-αklotho signalling as reflected by **(A)** a tendency towards elevated circulatory FGF23 levels in 5/6Nx mice and **(B)** progressively elevated FGF23 levels in ADE treated mice compared to control mice. Renal mRNA expression of FGFR1 and αKlotho in **(C)** CKD induced 5/6Nx mice (black bars) compared with sham-operated groups (white bars) and in **(D)** 2 weeks (black bars) and 4 weeks (gray bars) ADE treated mice compared with control groups (white bars). **(E)** Representative immunoblot (all 3 groups are done in one blot) and **(F)** semi-quantitative analysis for αKlotho **(E**; upper panel) and β-actin **(E**; lower panel) protein expression in renal lysates of control or ADE treated mice, which indicates reduced renal αKlotho protein expression upon CKD. Data are mean ± SEM. *: p<0.05 compared to control mice.

In order to determine whether induced kidney failure results in altered FGF23-αklotho signalling, we first measured blood FGF23 levels. FGF23 levels tended to be elevated in 5/6Nx mice when compared to sham-operated mice (Fig 2A), and progressively increased in ADE mice compared to control mice (Fig 2B; P<0.05). 5/6Nx did not affect renal mRNA expression of the primary FGF23 receptor FGFR1, while αklotho expression tended to be decreased, although not statistically significant (Fig 2C). ADE-treated mice demonstrated a mild raise in FGFR1 (after 4 weeks) and a progressive (>2-fold) reduction in renal αklotho mRNA expression (Fig 2D; P<0.05 for both time points) when compared to control mice. In addition, a 2-fold decline in renal αklotho expression following ADE treatment was confirmed on protein level (Fig 2E and 2F; P<0.05 for both time points).

Since inflammation is another hallmark of CKD we additionally checked whether an acute inflammatory stimulus affects FGF23 levels directly. Interestingly, both ConA and TNF administration *in vivo* resulted in significantly elevated renal FGF23 mRNA expression in WT mice (S1A Fig; P<0.05).

### CKD progression augmented both renal vitamin-D synthesis and blood PTH

**Fig 3:**
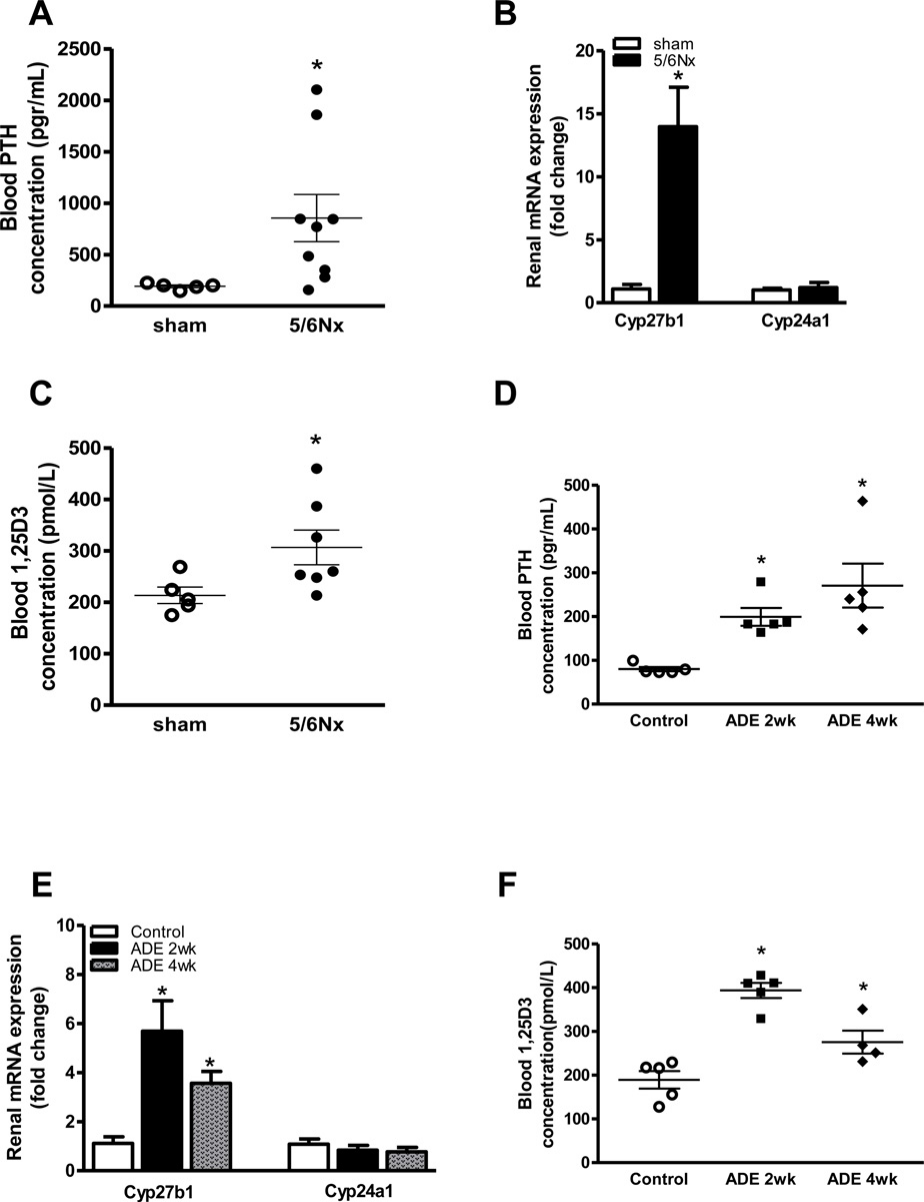
Experimental kidney failure augmented renal vitamin-D synthesis and induced secondary hyperparathyroidism. **(A)** PTH levels were elevated three weeks after 5/6Nx. **(B)** mRNA expression of Cyp27b1 and Cyp24a1 in 5/6Nx mice (black bars) compared with sham-operated group (white bars). **(C)** levels of 1,25-dihydroxy vitamin D_3_ in the blood were measured in mice with 5/6Nx nephrectomy. **(D)** PTH levels were elevated progressively in ADE treated mice in both 2 weeks and 4 weeks ADE treated mice compared with control groups. **(E)** mRNA expression of Cyp27b1 and Cyp24a1 in CKD induced by 2 weeks (black bars) and 4 weeks (gray bars) ADE treated mice compared with control groups (white bars) and **(F)** levels of 1,25-dihydroxy vitamin D_3_ increased in 2 weeks ADE treated mice in compare to control mice. This was significantly reduced in 4 weeks ADE treated mice in compare to 2 weeks ADE treated mice. Data are mean ± SEM. *: p<0.05 compared to either sham-operated or control mice.

CKD induced by either 5/6Nx or ADE-treatment augmented blood PTH levels in both 5/6Nx (Fig 3A; P<0.05) and ADE-treated mice (Fig 3D; P<0.05 for both time points). CKD development resulted in a 5- to 13-fold increased renal mRNA expression of the primary vitamin-D regulatory enzyme 25-hydroxyvitaminD_3_ 1α-hydroxylase (Cyp27b1) when compared to sham-operated or control-diet fed mice, respectively (Fig 3B and 3E). In contrast, no effects were observed on renal 1,25-dihydroxyvitamin D_3_ 24-hydroxylase (Cyp24a1) mRNA expression. In accordance, blood 1,25-dihydroxy vitamin D_3_ was higher in 5/6Nx mice compared to sham-operated mice after 3 weeks (Fig 3C; p<0.05), and was as well elevated in elevated in 2 and 4 weeks ADE-treated mice compared to control mice (Fig 3F; P<0.05 for both time points). However 4 weeks ADE-treated mice exhibited a significant reduction in the level of 1,25-dihydroxy vitamin D_3_ in compare to 2 weeks ADE-treated mice (Fig 3F; P<0.05; 4 weeks vs. 2 weeks).

### CKD altered expression of crucial renal calcium and phosphate transporters

**Fig 4:**
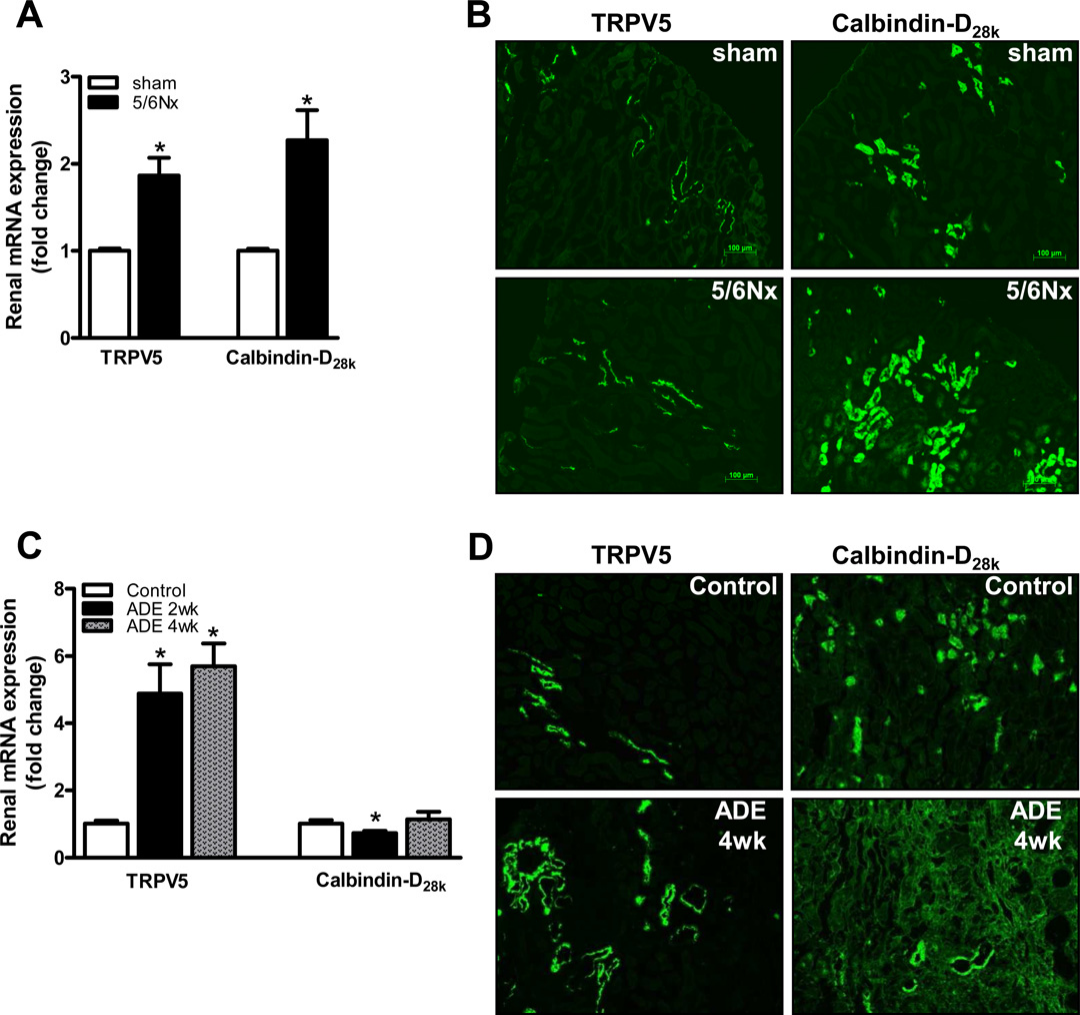
CKD development increased expression of tubular calcium transporters. **(A)** mRNA expression of the renal apical Ca^2+^ transporter TRPV5 and intracellular calcium shuttling protein calbindin-D_28K_ in the CKD induced 5/6Nx mice (black bars) compared to sham-operated mice (white bars). **(B)** Representative microphotographs for renal TRPV5 and calbindin-D_28K_ protein expression in kidney tissue from CKD induced 5/6Nx mice and sham-operated mice. **(C)** mRNA expression of the renal apical Ca^2+^ transporter TRPV5 and intracellular calcium shuttling protein calbindin-D_28K_ in mice fed with ADE diet for 2 weeks (black bars) and 4 weeks (gray bars) compared with control diet fed mice (white bars). **(D)** Representative microphotographs for renal TRPV5 and calbindin-D_28K_ protein expression in kidney tissue from CKD induced ADE treated (4 weeks) and control mice. Bars represent 100 μm. Data are mean ± SEM. *: p<0.05 compared to either sham-operated or control mice.

To characterize the impact of CKD on tubular electrolyte transport regulation, the renal expression of Ca^2+^ and P_i_ transporters was determined. Renal mRNA expression of TRPV5 and calbindin-D_28k_, which are specifically expressed in the distal convoluted tubule, was 2-fold increased in 5/6Nx mice compared to sham-operated mice (Fig 4A). In line, microphotographs of kidney tissue demonstrated concomitant increases on protein expression (Fig 4B). Additionally, ADE treatment induced a 5-fold increase of renal TRPV5, but did not affect renal calbindin-D_28k_ mRNA expression compared to control mice (Fig 4C). Comparable patterns were also observed on protein level (Fig 4D).

**Fig 5:**
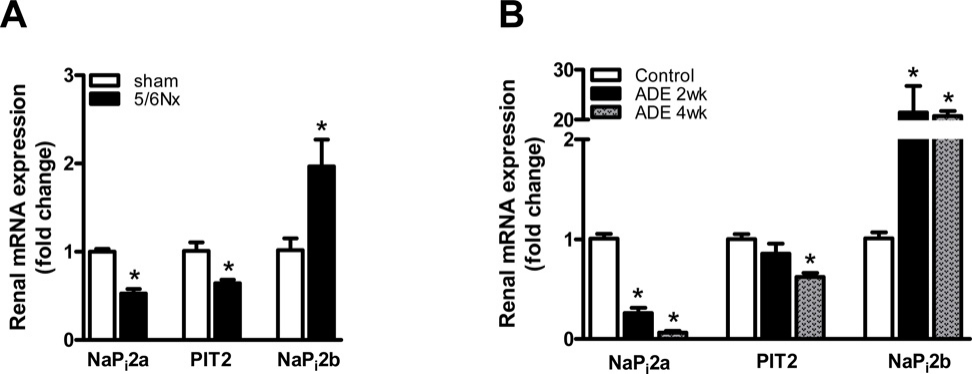
CKD development altered renal expression of tubular phosphate transporters. **(A)** mRNA expression of the renal NaP_i_2a, PIT2 and NaP_i_2b in mice with CKD development induced by either 5/6Nx (black bars) or **(B)** 2 weeks (black bars) and 4 weeks (gray bars) ADE treated mice ADE treatment, when compared to sham-operated (white bars) or control mice (white bars), respectively. Data are mean ± SEM. *: p<0.05 compared to either sham-operated or control mice.

Kidney injury induced by either 5/6Nx or ADE treatment reduced renal expression of sodium-dependent P_i_ transporters NaP_i_2a and PIT2 more than 2-fold, but elevated renal NaP_i_2b expression 2- to 20-fold compared to sham-operated or control mice, respectively (Fig 5A and 5B). On the contrary, renal expression of the thiazide-sensitive sodium-chloride co-transporter (NCC) specifically present in the distal convoluted tubule, was not affected in either 5/6Nx or ADE-treated mice (Data not shown).

### FGF23 initiated adaptive mechanisms of renal calcium handling

**Fig 6:**
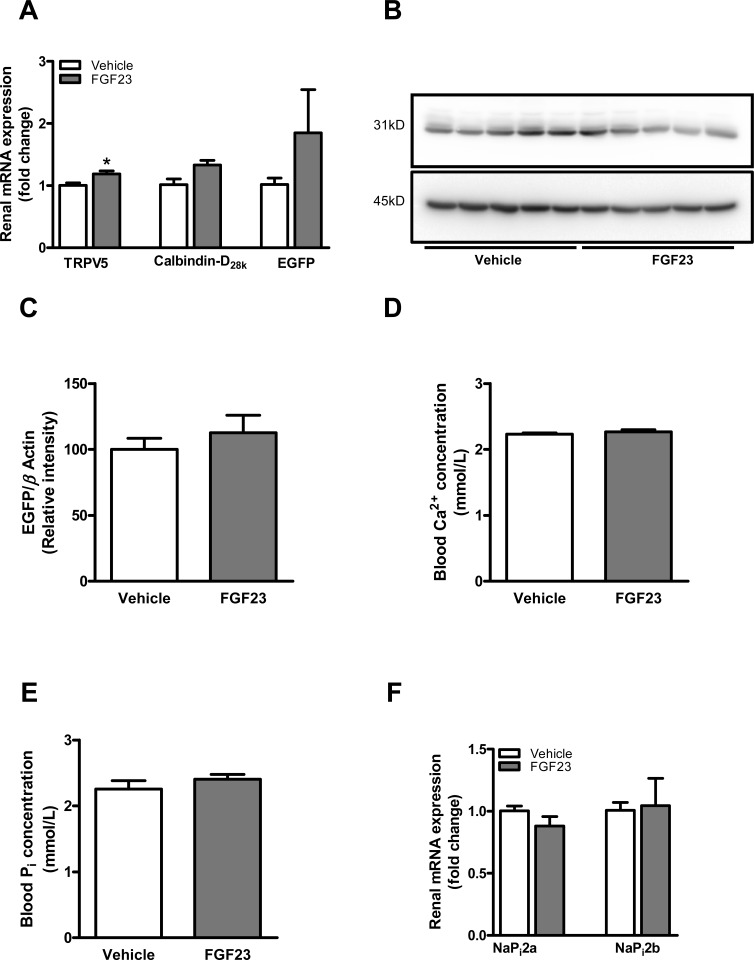
FGF23 administration directly affected renal tubular calcium transport. **(A)** Intravenous administration of recombinant FGF23 protein (dark-gray bars) results in increased TRPV5 and a tendency towards elevated calbindin-D_28k_ renal mRNA expression, when compared to vehicle-treated mice (white bars). Renal EGFP mRNA expression, as a surrogate marker for TRPV5 transcription tended to be increased following FGF23 administration compared to vehicle-treated mice. **(B)** Representative immunoblot and **(C)** semi-quantitative analysis of EGFP **(B**; upper panel) and β-actin **(B**; lower panel) protein expression in renal lysates of vehicle-treated or FGF23 injected mice. Blood **(D)** Ca^2+^ and **(E)** phosphate levels were measured following 24 hrs of FGF23 injection. **(F)** mRNA expression of the renal NaP_i_2a and NaP_i_2b in mice injected with FGF23 (gray bars), compared to vehicle-treated mice (white bars). Data are mean ± SEM. *: p<0.05 compared to control mice.

To gain insight whether inflammatory stimuli directly affect renal Ca^2+^ handling, TRPV5 mRNA expression was determined in WT mice following ConA or TNF administration. ConA, but not TNF administration resulted in elevated renal TRPV5 mRNA expression (S1B Fig). Furthermore, to determine whether FGF23 affects renal Ca^2+^ handling, total kidney tissue of transgenic mice, expressing EGFP driven by the TRPV5 promoter, was used to determine mRNA expression of TRPV5, calbindin-D_28k_ and EGFP, as a surrogate marker for TRPV5 transcription. Exogenous FGF23 administration resulted in an increased renal mRNA expression of TRPV5 (Fig 6A; P<0.05) and a tendency towards increased calbindin-D_28k_ expression (Fig 6A). In addition, EGFP mRNA expression tended to be elevated following FGF23 infusion, although not statistically significant, when compared to control mice (Fig 6A).

TRPV5 protein expression cannot be determined by immunoblotting directly because of the lack of appropriate primary antibodies. We therefore performed Western blotting on kidney lysates from transgenic mice that expresses Enhanced Green Fluorescent Protein (EGFP 31 kD) driven by the TRPV5 promoter. Using this approach, determination of EGFP act as a surrogate marker for TRPV5 expression. Representative pictures are shown in the Fig 6B. Digital quantification analysis showed no significant differences in GFP protein expression level between two groups (Fig 6C).

Ca^2+^ levels in the blood were unaffected after 24 hrs following FGF23 administration (Fig 6D). Changes in systemic phosphate levels in comparison to the control group were not detected. This might be due to the short-term period of the experiment. Unfortunately, we did not include the use of metabolic cages in the initial experimental setting. Therefore, the main effect of FGF23 infusion (P_i_ excretion) cannot be validated completely. In order to determine the local renal effects of rFGF23 infusion, gene expression of sodium-phosphate cotransporter type 2 (NaP_i_2a and NaP_i_2b) in renal tissue were measured. No alterations between the groups were however observed (Fig 6F).

## Discussion

Ca^2+^ and P_i_ disturbances are a hallmark of CKD and an important risk factor for the development of severe cardiovascular complications. In the current study, we characterized the impact of CKD on systemic and local renal factors involved in Ca^2+^ and P_i_ regulation. Our data reveal that two different experimental models of kidney failure induce comparable classical features of CKD. A deregulated Ca^2+^ and P_i_ homeostasis and a significantly disturbed FGF23-αklotho-vitamin-D axis were observed in both models. Both models resulted in an increased or decreased expression of specific renal Ca^2+^ and P_i_ transporters, respectively.

First, both models of kidney failure, that is 5/6Nx and ADE treatment, induced clinical hallmarks of CKD, as reflected by the development of uraemia and an elevated renal expression of tubular injury marker NGAL. Our results are in accordance with previous studies demonstrating that both partial nephrectomy [31–33] and ADE treatment [34–36] closely resemble various features of CKD development. The two models we used gave rise to different severity in terms of its consequences on systemic urea and NGAL, electrolyte disturbances and alterations of the FGF23-αklotho-vitamin-D axis, all pointing to more severe damage in the ADE-model.

Second, both experimental models disturbed Ca^2+^ homeostasis comparably, as reflected by hypercalcemia and elevated urinary Ca^2+^ excretion. Hypercalciuria as a consequence of partial nephrectomy was shown previously [37], and can be explained by increased filtration burden on remnant glomeruli causing increased ion excretion and parallel development of renal osteodystrophy [38]. The hypercalcemia observed in both models is likely due to early elevated PTH levels and aggravated by 1,25-dihydroxy vitamin-D_3_-facilitated intestinal Ca^2+^ absorption. In contrast to our data, hypercalcemia was not observed following ADE treatment in mice [39, 40], although this might be explained by variation in the amount of adenine supplemented to the diet, and thus in degree of renal injury induced. While ADE treatment increased blood urea levels to ~60 mmol/L after 4 weeks in this study, other studies showed maximal urea levels of around 40 mmol/L, suggesting a distinct degree of renal injury. In agreement with previous studies [36, 39, 40], ADE treatment dramatically affected P_i_ homeostasis, a common hallmark observed in severe CKD [41]. ADE mice presented a significant rise on P_i_ levels in the blood despite that elevated fractional excretion was initially adequate to manage P_i_ loading. In contract, no effects on systemic P_i_ levels were detected three weeks after 5/6Nx, suggesting that modest elevated levels of PTH and FGF23 are sufficient to maintain physiological blood P_i_ levels.

Third, both experimental models were characterized by a deregulated FGF23-αklotho-vitamin-D axis. We observed an increase in systemic FGF23 levels and a reduction in renal αklotho expression. Previously it has been suggested that lack of αklotho might be a driving force for FGF23 resistance [42–48]. It is, however, not yet clear whether the downregulation of αklotho can trigger an increase in the expression of FGF23 or vice versa. On the one hand, FGF23 appears to be an early marker of CKD and rises progressively with progression of CKD. On the other hand, loss of renal αklotho occurs early in CKD, and might be the trigger to increase systemic FGF23 in order to maintain P_i_ homeostasis. Additionally, rising FGF23 levels upon CKD might also be due to inflammation [49, 50]. Indeed, it has been shown that both experimental models significantly induce renal cytokine levels [51, 52] and recent studies point out that elevated blood FGF23 levels are associated with inflammatory components (e.g. cytokines/chemokines) [53–55], and in CKD patients FGF23 and inflammatory markers are often both increased [55, 56]. Interestingly, a study by Mendoza *et al*., [57] showed that higher circulatory levels of FGF23 directly correlate with increased blood levels of several proinflammatory markers, suggesting that FGF23 is independently associated with inflammation in CKD. In line, we also observed that two different inflammatory stimuli acutely induce renal FGF23 mRNA expression. Although, in the current study we did not further elucidate the effects of inflammatory pathways on electrolyte regulatory hormones upon kidney failure, as this was beyond the scope of the primary project.

Despite elevated blood FGF23, both kidney failure models augmented renal Cyp27b1 expression, probably due to the observed increase in PTH. Comparable data were shown before in remnant kidneys of nephrectomized rats [58], and following ADE-treatment [59]. In accordance, elevated blood 1,25-dihydroxy vitamin D_3_ levels were detected following both 5/6Nx and ADE treatment. Together, these findings indicate that damaged kidneys retain sufficient vitamin-D synthesizing capacity and that an increased Cyp27b1 expression in the remaining epithelium might be effective to compensate for the loss of tissue capable of Cyp27b1 expression due to the reduction in functional kidney mass. Moreover, FGF23-suppressive effects on 1,25-dihydroxy vitamin D_3_ either directly via modulation of renal Cyp27b1 and Cyp24a1 expression, or indirectly via modulation of PTH synthesis might be hampered due to lower renal αklotho expression in both kidneys and parathyroid gland, implicating FGF23 resistance [60–62]. As a proposed pathway, this points towards early high PTH levels as the likely mechanism responsible for amplifying renal vitamin-D synthesis and the consequent rise in blood 1,25-dihydroxy vitamin D_3_ and Ca^2+^ levels. Indeed, both CKD models were characterized by the development of secondary hyperparathyroidism. Nevertheless, this was not in line with the observed hypercalcemia, elevated levels of FGF23 and increase in 1,25-dihydroxy vitamin D_3_, which all should have suppress PTH level. However, we did not examine parathyroid tissue to study local changes that may drive PTH production or secretion. Development of secondary hyperparathyroidism could be due to early disturbances on the local calcium sensing receptor, early decline in parathyroid klotho and possibly lower expression of the vitamin D receptor.

Fourth, to identify local adaptive mechanisms of tubular electrolyte regulation following CKD, renal expression of crucial Ca^2+^ and P_i_ transporters was determined. Gene expression of Ca^2+^ transporters TRPV5 and calbindin-D_28k_ was elevated upon CKD. TRPV5 is a crucial rate-limiting Ca^2+^ channel expressed in the late distal convoluted and connecting tubule, responsible for apical Ca^2+^ influx initiating transcellular reabsorption [63, 64]. Calbindin-D_28k_ is subsequently responsible for the direct intracellular Ca^2+^ shuttling towards the basolateral extrusion side [65]. Together, this suggests that elevated distal tubular Ca^2+^ reabsorption is likely the consequence of stimulation by calciotropic factors, including PTH, vitamin D and/or FGF23 signalling [28, 66, 67]. However, Calbindin-D_28k_ levels were differentially altered in the ADE dietary intervention model; we observed either reduction or no changes in the Calbindin-D_28k_ gene expression levels, while it increased in the 5/6Nx model. The reason for that is yet unclear but it could be that adenine itself might affect the expression/regulation of intracellular (Ca^2+^) proteins, that result in a different kinetics compared to TRPV5. In order to determine whether FGF23 directly affects renal Ca^2+^ transport, we administered FGF23 to TRPV5-EGFP transgenic mice to verify its effect on renal EGFP expression, as a surrogate marker for TRPV5 transcription. We found, at most, a tendency towards increased renal EGFP expression. In contrast, a previous study indicated a direct effect of FGF23 on renal Ca^2+^ transport by increasing TRPV5 gene transcription [68]. A potential explanation for this discrepancy might be that essential upstream enhancer elements for the transcriptional induction of TRPV5 by FGF23 are lacking in the TRPV5-EGFP mice. Interestingly, WT mice exposed to an inflammatory stimulus (ConA, but not TNF treatment) demonstrate elevated renal TRPV5 mRNA expression. Together, these results indicate that both hormonal and inflammatory parameters may influence renal Ca^2+^ transport, although the exact stimuli and signalling pathways should be further investigated in future studies.

In concert with the phosphaturic effects of FGF23 and PTH [69], both kidney failure models displayed a reduced renal expression of NaP_i_2a and PIT2, the principal renal type II and III sodium-dependent P_i_ transporters, respectively [70–72]. NaP_i_2a is the main P_i_ co-transporter involved in the phosphaturic response to both PTH and FGF23 and it responded to high FGF23 as expected despite the observed reductions in renal αklotho. On the contrary, renal NaP_i_2b was significantly augmented. NaP_i_2b as a predominant intestinal P_i_ transporter is also expressed at the basolateral side of renal epithelial cells. Its expression is increased in response to active phosphate loading [73]. Hence NaP_i_2b plays major roles in both the kidney and the small intestine. Mice with NaP_i_2b deficiency, exhibited predominantly lower P_i_ absorption [74], highlighting a major role of NaP_i_2b in body P_i_ homeostasis. As proposed, elevated renal NaP_i_2b expression might be an additional mechanism of active tubular P_i_ secretion, although it remains unclear whether this also holds true during progressive renal injury to counteract P_i_ retention. Since 5/6Nx and ADE treatment induced distinct renal pathophysiological mechanisms, its consequences on renal expression of the sodium-chloride co-transporter (NCC) were determined. NCC expression was unaffected within both models, suggesting that the demonstrated effect on the expression kinetics of Ca^2+^ and P_i_ transporters within both models was not simply a reflection of distinct kidney tissue composition compared to sham-operated or control mice as a potential confounding factor.

Interestingly, the increased blood Ca^2+^ and active 1,25-dihydroxy vitamin D_3_ observed in the current study do not correspond with the classical observation of hypocalcemia due to reduced 1,25-dihydroxy vitamin D_3_ levels that are frequently observed in clinically diagnosed early stage renal disease patients. The reason for this is unclear but it might be related to the timing of the measurements performed in our experimental models. This is possibly demonstrated by the 1,25-dihydroxy vitamin D_3_ levels observed in the ADE mice model, which is increased on week 2, and reduced on week 4. Here we present an early renal failure model, possibly not at steady state yet. Moreover, differences between species may explain why a given response in animal models differs from patients with CKD [75]. Furthermore, several physiological differences between experimental animal models and patients can be identified, including variations in time to develop a certain degree of kidney disease reflecting a particular stage of CKD. Here, we observed hypercalcemia and hypercalciuria in both experimental CKD mice models (Table 2) and these observations are supported by previously applied experimental CKD animal models [76], but not in patients with CKD [77–79]. Human CKD development reflects a gradual and slow decline of vitamin D levels, contributing to hypocalcemia, whereas secondary hyperparathyroidism develops over longer time. On the contrary, experimental animal models are rather short term, due to the methods applied and the shorter lifespan of rodents [80]. One of the limitations of this study is that we did not keep both models for a longer period of time. Furthermore, CKD-induced alterations in intestinal and/or bone Ca^2+^ and P_i_ homeostasis might also trigger an increase in blood Ca^2+^ and active 1,25-dihydroxy vitamin D_3_ observed in these models. Yet, here we primarily focused on renal Ca^2+^ and P_i_ regulatory mechanisms; hence the existing experiment was not designed to characterize the impact of CKD-induced alterations on intestinal Ca^2+^ and P_i_ transport or bone metabolism. It thus remains difficult to fully elucidate the compensatory mechanisms occurring following CKD, which might contribute to the disturbed electrolyte homeostasis observed.

Taken together, our study demonstrated that murine models of partial nephrectomy and ADE-enriched dietary treatment, both, induce kidney failure that closely resembles CKD development, which is associated with disturbed FGF23-αklotho-vitamin-D signalling and a deregulated Ca^2+^ and P_i_ homeostasis. Moreover, this study identified local tubular adaptive mechanisms that are involved in disturbed Ca^2+^ and P_i_ regulation, hence enabling new opportunities to target mineral disturbances emerging as a consequence of CKD development.

## Supporting Information

S1 Table Primer sequences used for real-time quantitative RT-PCR. (DOCX)

S1 Fig The effects on renal FGF23 and TRPV5 mRNA expression in mice injected with inflammatory stimuli ConA and TNF. **(A)** Renal mRNA expression of FGF23 or **(B)** TRPV5 in mice injected with ConA (left graph) and TNF (right graph), both compared with vehicle-treated mice (PBS). Data are indicated as fold induction and present a mean ± SEM. *: p<0.05 compared to control mice.
(TIF)

S1 File The ARRIVE Checklist. (PDF)
