# Supplementary material for: Deregulated Renal Calcium and Phosphate Transport during Experimental Kidney Failure
Source: PLoS One. 2015 Nov 13;10(11):e0142510. doi: 10.1371/journal.pone.0142510 (PMC4643984; doi:10.1371/journal.pone.0142510)
Supplement: S1 Table — (DOCX) [file pone.0142510.s001.docx]

## Supporting Information

**S1 Table: Primer sequences used for real-time quantitative RT-PCR**

| Gene | Forward primer 5’-3’ | Reverse primer 5’-3’ |
| --- | --- | --- |
| ***HPRT*** | TTGCTGACCTGCTGGATTAC | AGTTGAGAGATCATCTCCAC |
| ***FGF23*** | TATGGATCTCCACGGCAAC | GTCCACTGGCGGAACTTG |
| ***Klotho*** | GGTTGCCCACAACCTACTTT | TGGGAGCTTAAGGCGATAGA |
| ***TRPV5*** | CTCCGCCTTCCATCGAAGTTC | GATGTCAGCTCCATGCTCAATG |
| ***Calbindin-D_28k_*** | GACGGAAGTGGTTACCTGGA | ATTTCCGGTGATAGCTCCAA |
| ***Cyp27b1*** | GTGTTGAGATTGTACCCTGTGG | TGGGGAATTACATAGTTTCCTACAC |
| ***Cyp24a1*** | GGAGTCCATGAGGCTTACCC | GGTAGCGTGTATTCACCCAGA |
| ***NaP_i_2a*** | TCAGGAAGAGGAGCAAAAGC | AAAGGAAAGCCAGCATCAGA |
| ***NaP_i_2b*** | CTATTCCGCCCTGGTTCTC | GAAAATGCAGAGCGTCTTCC |
| ***FGFR1*** | TAAGATCGGGCCAGACAACT | CGATAGAGTTACCCGCCAAG |
| ***NGAL*** | CTTCTCTGTCCCCACCGACCAATG | GACAGACAGGCAGCGCCTGAA |
| ***PIT2*** | CGGCGTGCTGTTCATACTAA | GCAGCATAAAACAGAGGCAGT |
| ***NCC*** | CTTCGGCCACTGGCATTCTG | GATGGCAAGGTAGGAGATGG |
| ***EGFP*** | GCAGAAGAACGGCATCAAG | TGCTCAGGTAGTGGTTGTCG |

HPRT, hypoxanthine-guanine phosphoribosyl transferase; FGF23, Fibroblast Growth Factor 23; Klotho, αklotho; TRPV5, transient receptor potential cation channel subfamily V member 5; Calbindin-D_28k_, calbindin-D_28k_; Cyp27b1, 25-hydroxyvitamin D_3_ 1α-hydroxylase; Cyp24a1, 1,25-dihydroxyvitamin D_3_ 24-hydroxylase; NaP_i_2a, sodium-phosphate cotransporter type 2a; NaP_i_2b, sodium-phosphate cotransporter type 2b; FGFR1, fibroblast growth factor receptor type-I;NGAL, neutrophil gelatinase-associated lipocalin; PIT2, sodium-dependent P_i_ transporter type 3; NCC, sodium-chloride cotransporter; EGFP, enhanced green fluorescence protein.
