# Supplementary figures and images for: Deregulated Renal Calcium and Phosphate Transport during Experimental Kidney Failure
Source: PLoS One. 2015 Nov 13;10(11):e0142510. doi: 10.1371/journal.pone.0142510 (PMC4643984; doi:10.1371/journal.pone.0142510)

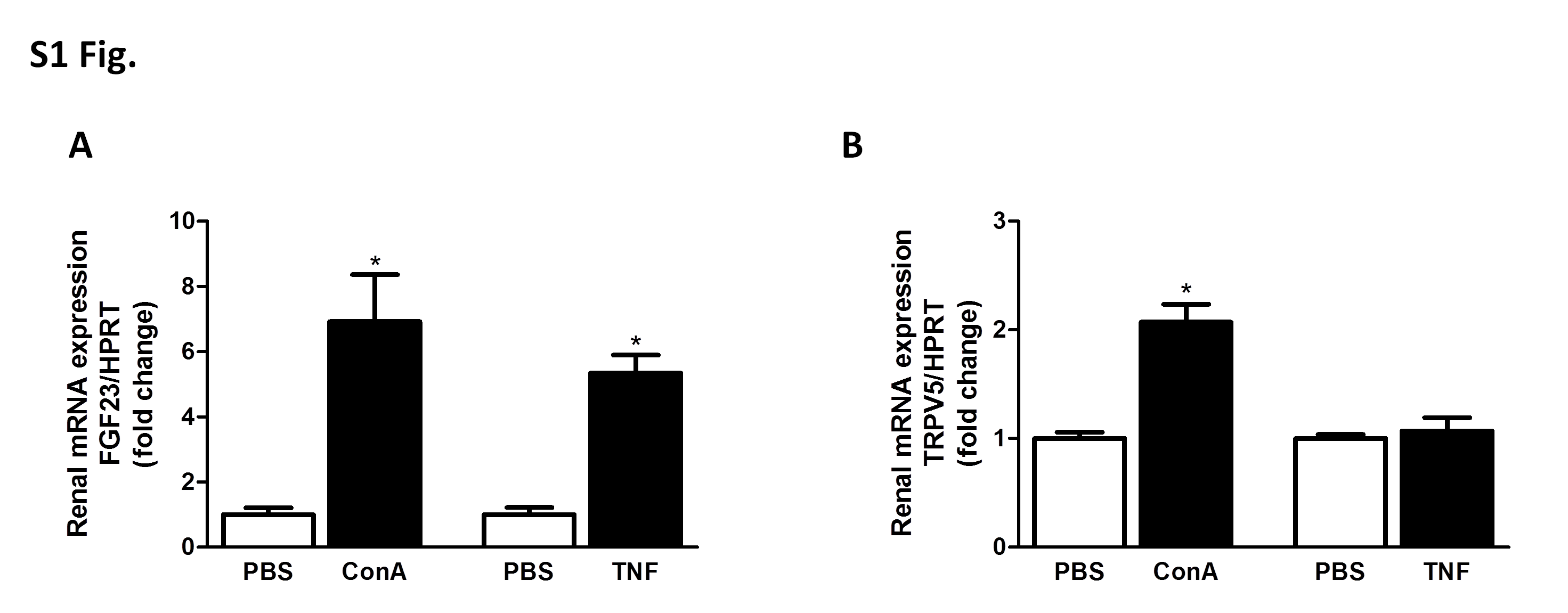

Supplement: S1 Fig — (A) Renal mRNA expression of FGF23 or (B) TRPV5 in mice injected with ConA (left graph) and TNF (right graph), both compared with vehicle-treated mice (PBS). Data are indicated as fold induction and present a mean ± SEM. *: p<0.05 compared to control mice. (TIF) [file pone.0142510.s002.TIF]
